# Dibromido(2,3,5,6-tetra-2-pyridyl­pyrazine-κ^3^ *N* ^2^,*N* ^1^,*N* ^6^)zinc(II)

**DOI:** 10.1107/S1600536810027820

**Published:** 2010-07-17

**Authors:** Roya Ahmadi, Khadijeh Kalateh, Vahid Amani

**Affiliations:** a Islamic Azad University, Shahr-e-Rey Branch, Tehran, Iran

## Abstract

In the title compound, [ZnBr_2_(C_24_H_16_N_6_)], the Zn^II^ ion is coordinated by the *N*,*N*′,*N*′′-tridentate 2,3,5,6-tetra-2-pyridyl­pyrazine ligand and two bromide ions, generating a distorted ZnN_3_Br_2_ trigonal-bipyramidal geometry for the metal ion, with both bromide ions in equatorial sites. The dihedral angles between the pyrazine ring and the coordinated pyridine rings are 13.3 (2) and 24.8 (2)°; those between the pyrazine ring and the uncoordinated pyradine rings are 31.3 (2) and 44.2 (2)°. In the crystal, inversion dimers linked by pairs of weak C—H⋯Br hydrogen bonds occur.

## Related literature

For the synthesis of the ligand, see: Goodwin & Lyons (1959 ▶). For the structure of the free ligand, see Bock *et al.* (1992 ▶); Greaves & Stoeckli-Evans (1992 ▶). For related structures, see: Alizadeh *et al.* (2009 ▶); Carranza *et al.* (2004 ▶); Graf *et al.* (1993 ▶, 1997 ▶); Hadadzadeh *et al.* (2006 ▶); Laine *et al.* (1995 ▶); Morsali & Ramazani (2005 ▶); Sakai & Kurashima (2003 ▶); Seyed Sadjadi *et al.* (2008 ▶); Yamada *et al.* (2000 ▶); Zhang *et al.* (2005 ▶).
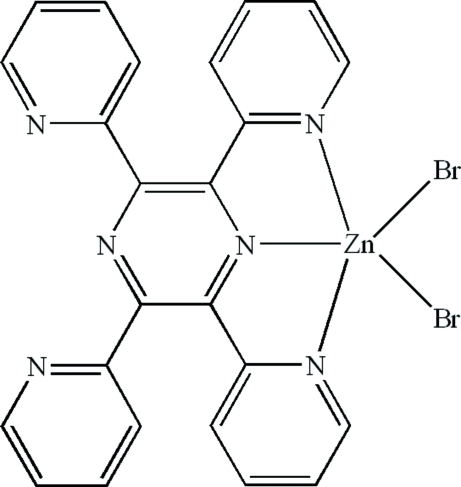


## Experimental

### 

#### Crystal data

[ZnBr_2_(C_24_H_16_N_6_)]
*M* *_r_* = 613.62
Triclinic, 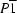

*a* = 10.3985 (8) Å
*b* = 10.5378 (8) Å
*c* = 12.3034 (10) Å
α = 64.898 (6)°
β = 83.187 (6)°
γ = 77.901 (6)°
*V* = 1193.05 (16) Å^3^
*Z* = 2
Mo *K*α radiation
μ = 4.40 mm^−1^
*T* = 298 K
0.50 × 0.40 × 0.28 mm

#### Data collection

Bruker SMART CCD diffractometer
Absorption correction: multi-scan (*SADABS*; Bruker, 1998 ▶) *T*_min_ = 0.206, *T*_max_ = 0.369
13823 measured reflections
6412 independent reflections
4954 reflections with *I* > 2σ(*I*)
*R*_int_ = 0.049

#### Refinement

*R*[*F* ^2^ > 2σ(*F* ^2^)] = 0.051
*wR*(*F* ^2^) = 0.126
*S* = 1.14
6412 reflections
298 parameters
H-atom parameters constrained
Δρ_max_ = 0.89 e Å^−3^
Δρ_min_ = −0.91 e Å^−3^

### 

Data collection: *SMART* (Bruker, 1998 ▶); cell refinement: *SAINT* (Bruker, 1998 ▶); data reduction: *SAINT*; program(s) used to solve structure: *SHELXTL* (Sheldrick, 2008 ▶); program(s) used to refine structure: *SHELXTL*; molecular graphics: *SHELXTL*; software used to prepare material for publication: *WinGX* (Farrugia, 1999 ▶).

## Supplementary Material

Crystal structure: contains datablocks I, global. DOI: 10.1107/S1600536810027820/hb5552sup1.cif

Structure factors: contains datablocks I. DOI: 10.1107/S1600536810027820/hb5552Isup2.hkl

Additional supplementary materials:  crystallographic information; 3D view; checkCIF report

## Figures and Tables

**Table:** 

Zn1—N2	2.151 (3)
Zn1—N6	2.177 (3)
Zn1—N1	2.202 (3)
Zn1—Br1	2.3692 (7)
Zn1—Br2	2.3880 (6)

**Table:** 

N2—Zn1—N6	73.75 (10)
N2—Zn1—N1	72.96 (11)
N6—Zn1—N1	146.71 (11)
N2—Zn1—Br1	125.38 (8)
N6—Zn1—Br1	102.12 (9)
N1—Zn1—Br1	97.43 (10)
N2—Zn1—Br2	118.61 (8)
N6—Zn1—Br2	97.55 (9)
N1—Zn1—Br2	97.76 (10)
Br1—Zn1—Br2	115.93 (2)

**Table 2:** Hydrogen-bond geometry (Å, °)

*D*—H⋯*A*	*D*—H	H⋯*A*	*D*⋯*A*	*D*—H⋯*A*
C11—H11⋯Br2^i^	0.93	2.88	3.791 (7)	166

Symmetry code: (i) 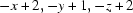.

**Fig. 1.**
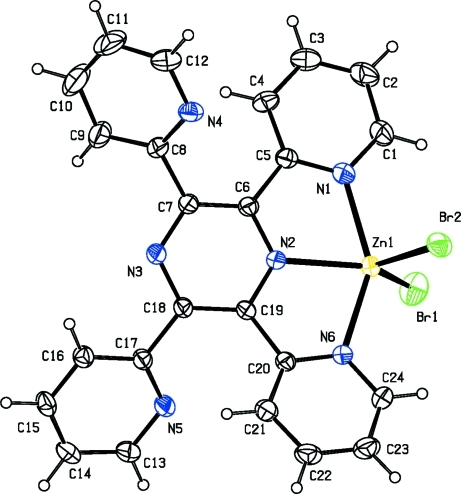
The molecular structure of (I) with displacement ellipsoids drawn at the 30% probability level.

**Fig. 2.**
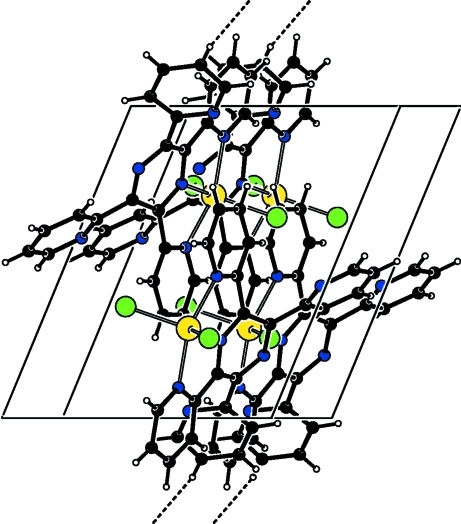
Unit-cell packing diagram for (I).

## supplementary crystallographic information

### Comment

Goodwin & Lyons (1959) reported the synthesis of 2,3,5,6-tetra(2-pyridinyl)pyrazine (tppz). Bock *et al.* (1992) and Greaves & Stoeckli-Evans (1992) determined the structure of tppz by single-crystal X-ray diffraction methods. tppz is a good bis-tridentate bridging ligand, and numerous complexes with tppz have been prepared, such as that of ruthenium (Hadadzadeh *et al.*, 2006), platinum (Sakai & Kurashima, 2003), mercury (Zhang *et al.*, 2005), copper (Carranza *et al.*, 2004), iron (Laine *et al.*, 1995), nickel (Graf *et al.*, 1997), palladium (Yamada *et al.*, 2000), cadmium (Seyed Sadjadi *et al.*, 2008) and lead (Morsali & Ramazani, 2005). For further investigation of 2,3,5,6-tetra(2-pyridinyl)pyrazine, we synthesis the title complex, (I), and report herein its crystal structure.

In the title compound, (Fig. 1), the Zn^II^ atom is five-coordinated in a distorted trigonal-bipyramidal configuration by three N atoms from one 2,3,5,6-tetra(2-pyridinyl)pyrazine and two terminal Br. The Zn—N and Zn—Br bond lengths and angles (Table 1) are within normal range of [ZnCl_2_ (tppz)], (Graf *et al.*, 1993) and [ZnBr_2_(6,6'-dmbpy)], (Alizadeh *et al.*, 2009) [where 6,6'-dmbpy is 6,6'-dimethyl-2, 2'-bipyridine] respectively.

In the crystal structure, intermolecular C—H···Br hydrogen bonds (Table 2, Fig. 2) may stabilize the structure.

### Experimental

A solution of 2,3,5,6-tetra(2-pyridinyl)pyrazine (0.40 g, 1.00 mmol) in HCCl_3_ (20 ml) was added to a solution of ZnBr_2_ (0.23 g, 1.00 mmol) in methanol (20 ml) at room temperature. The suitable crystals for X-ray diffraction experiment were obtained by methanol diffusion to a colorless solution in DMSO. Yellow prisms of (I) were isolated after one week (yield; 0.45 g, 73.3%).

### Refinement

All H atoms were positioned geometrically, with C—H = 0.93Å and constrained to ride on their parent atoms, with *U*_iso_(H) = 1.2*U*_eq_(C).

### Crystal data

**Table:**

[ZnBr_2_(C_24_H_16_N_6_)]	*Z* = 2
*M**_r_* = 613.62	*F*(000) = 604
Triclinic, *P*1	*D*_x_ = 1.708 Mg m^−^^3^
Hall symbol: -P 1	Mo *K*α radiation, λ = 0.71073 Å
*a* = 10.3985 (8) Å	Cell parameters from 998 reflections
*b* = 10.5378 (8) Å	θ = 1.8–29.3°
*c* = 12.3034 (10) Å	µ = 4.40 mm^−^^1^
α = 64.898 (6)°	*T* = 298 K
β = 83.187 (6)°	Prism, yellow
γ = 77.901 (6)°	0.50 × 0.40 × 0.28 mm
*V* = 1193.05 (16) Å^3^	

### Data collection

**Table:**

Bruker SMART CCD diffractometer	6412 independent reflections
Radiation source: fine-focus sealed tube	4954 reflections with *I* > 2σ(*I*)
graphite	*R*_int_ = 0.049
phi and ω scans	θ_max_ = 29.2°, θ_min_ = 1.8°
Absorption correction: multi-scan (*SADABS*; Bruker, 1998)	*h* = −14→14
*T*_min_ = 0.206, *T*_max_ = 0.369	*k* = −13→14
13823 measured reflections	*l* = −16→16

### Refinement

**Table:**

Refinement on *F*^2^	Primary atom site location: structure-invariant direct methods
Least-squares matrix: full	Secondary atom site location: difference Fourier map
*R*[*F*^2^ > 2σ(*F*^2^)] = 0.051	Hydrogen site location: inferred from neighbouring sites
*wR*(*F*^2^) = 0.126	H-atom parameters constrained
*S* = 1.14	*w* = 1/[σ^2^(*F*_o_^2^) + (0.0524*P*)^2^ + 0.8386*P*] where *P* = (*F*_o_^2^ + 2*F*_c_^2^)/3
6412 reflections	(Δ/σ)_max_ = 0.010
298 parameters	Δρ_max_ = 0.89 e Å^−^^3^
0 restraints	Δρ_min_ = −0.91 e Å^−^^3^

### Special details

**Table:**

Geometry. All e.s.d.'s (except the e.s.d. in the dihedral angle between two l.s. planes) are estimated using the full covariance matrix. The cell e.s.d.'s are taken into account individually in the estimation of e.s.d.'s in distances, angles and torsion angles; correlations between e.s.d.'s in cell parameters are only used when they are defined by crystal symmetry. An approximate (isotropic) treatment of cell e.s.d.'s is used for estimating e.s.d.'s involving l.s. planes.
Refinement. Refinement of *F*^2^ against ALL reflections. The weighted *R*-factor *wR* and goodness of fit *S* are based on *F*^2^, conventional *R*-factors *R* are based on *F*, with *F* set to zero for negative *F*^2^. The threshold expression of *F*^2^ > σ(*F*^2^) is used only for calculating *R*-factors(gt) *etc*. and is not relevant to the choice of reflections for refinement. *R*-factors based on *F*^2^ are statistically about twice as large as those based on *F*, and *R*- factors based on ALL data will be even larger.

### Fractional atomic coordinates and isotropic or equivalent isotropic displacement parameters (Å^2^)

**Table:**

	*x*	*y*	*z*	*U*_iso_*/*U*_eq_	
Zn1	0.69495 (4)	0.37649 (4)	0.71272 (4)	0.03753 (11)	
Br2	0.62972 (5)	0.62861 (4)	0.64284 (4)	0.05470 (13)	
Br1	0.52355 (5)	0.24651 (6)	0.74414 (5)	0.07140 (16)	
N5	1.1123 (3)	0.0445 (4)	0.5766 (3)	0.0452 (7)	
N3	1.1520 (3)	0.1584 (3)	0.7992 (3)	0.0397 (6)	
C7	1.0799 (3)	0.2101 (4)	0.8729 (3)	0.0379 (7)	
C20	0.9186 (3)	0.3265 (3)	0.5446 (3)	0.0352 (7)	
C16	1.3156 (3)	0.0461 (4)	0.6499 (3)	0.0397 (7)	
H16	1.3598	0.0784	0.6918	0.048*	
C6	0.9439 (3)	0.2551 (4)	0.8580 (3)	0.0350 (7)	
C17	1.1801 (3)	0.0874 (4)	0.6368 (3)	0.0363 (7)	
N6	0.7888 (3)	0.3729 (3)	0.5459 (3)	0.0407 (7)	
N2	0.8986 (3)	0.2850 (3)	0.7503 (2)	0.0339 (6)	
C8	1.1517 (4)	0.2208 (4)	0.9655 (3)	0.0423 (8)	
C19	0.9768 (3)	0.2574 (4)	0.6653 (3)	0.0341 (6)	
C5	0.8400 (4)	0.2731 (4)	0.9463 (3)	0.0377 (7)	
C18	1.1009 (3)	0.1719 (4)	0.6992 (3)	0.0353 (7)	
N4	1.1146 (3)	0.3429 (4)	0.9781 (3)	0.0457 (7)	
N1	0.7200 (3)	0.3285 (4)	0.9022 (3)	0.0475 (7)	
C13	1.1800 (4)	−0.0388 (5)	0.5255 (4)	0.0499 (9)	
H13	1.1346	−0.0669	0.4811	0.060*	
C15	1.3823 (4)	−0.0435 (4)	0.5994 (3)	0.0453 (8)	
H15	1.4724	−0.0751	0.6087	0.054*	
C21	0.9930 (4)	0.3513 (4)	0.4381 (3)	0.0448 (8)	
H21	1.0837	0.3210	0.4388	0.054*	
C3	0.7577 (5)	0.2571 (5)	1.1400 (4)	0.0567 (11)	
H3	0.7711	0.2326	1.2204	0.068*	
C23	0.7940 (5)	0.4678 (5)	0.3319 (4)	0.0584 (11)	
H23	0.7493	0.5150	0.2605	0.070*	
C12	1.1751 (5)	0.3588 (5)	1.0604 (4)	0.0592 (11)	
H12	1.1516	0.4439	1.0696	0.071*	
C1	0.6194 (5)	0.3486 (6)	0.9748 (4)	0.0646 (13)	
H1	0.5356	0.3851	0.9440	0.078*	
C22	0.9278 (5)	0.4225 (5)	0.3307 (3)	0.0525 (10)	
H22	0.9746	0.4396	0.2581	0.063*	
C9	1.2494 (5)	0.1128 (6)	1.0304 (5)	0.0731 (16)	
H9	1.2749	0.0302	1.0174	0.088*	
C24	0.7276 (4)	0.4421 (5)	0.4402 (4)	0.0548 (10)	
H24	0.6371	0.4735	0.4410	0.066*	
C2	0.6354 (5)	0.3171 (6)	1.0948 (4)	0.0660 (13)	
H2	0.5648	0.3364	1.1427	0.079*	
C11	1.2693 (7)	0.2565 (7)	1.1313 (6)	0.0866 (19)	
H11	1.3067	0.2706	1.1893	0.104*	
C10	1.3082 (7)	0.1332 (8)	1.1165 (7)	0.103 (3)	
H10	1.3737	0.0629	1.1635	0.123*	
C14	1.3150 (4)	−0.0862 (4)	0.5348 (4)	0.0486 (9)	
H14	1.3587	−0.1453	0.4983	0.058*	
C4	0.8616 (4)	0.2330 (5)	1.0660 (3)	0.0488 (9)	
H4	0.9450	0.1903	1.0964	0.059*	

### Atomic displacement parameters (Å^2^)

**Table:**

	*U*^11^	*U*^22^	*U*^33^	*U*^12^	*U*^13^	*U*^23^
Zn1	0.03347 (19)	0.0393 (2)	0.0397 (2)	0.00120 (15)	−0.00453 (14)	−0.01878 (17)
Br2	0.0683 (3)	0.0393 (2)	0.0545 (2)	0.00334 (18)	−0.00494 (19)	−0.02253 (18)
Br1	0.0635 (3)	0.0628 (3)	0.0887 (4)	−0.0252 (2)	−0.0112 (2)	−0.0232 (3)
N5	0.0386 (15)	0.0507 (18)	0.0572 (19)	0.0007 (13)	−0.0060 (13)	−0.0352 (16)
N3	0.0376 (14)	0.0409 (16)	0.0463 (16)	0.0017 (12)	−0.0089 (12)	−0.0254 (13)
C7	0.0416 (17)	0.0347 (16)	0.0412 (17)	0.0010 (13)	−0.0091 (14)	−0.0208 (14)
C20	0.0408 (17)	0.0304 (15)	0.0355 (16)	−0.0001 (13)	−0.0045 (13)	−0.0167 (13)
C16	0.0341 (16)	0.0428 (19)	0.0438 (18)	−0.0068 (14)	0.0001 (13)	−0.0196 (15)
C6	0.0415 (17)	0.0304 (15)	0.0329 (15)	−0.0010 (13)	−0.0051 (12)	−0.0143 (13)
C17	0.0326 (15)	0.0370 (17)	0.0432 (17)	−0.0034 (13)	−0.0016 (13)	−0.0213 (14)
N6	0.0410 (15)	0.0462 (17)	0.0352 (14)	0.0037 (13)	−0.0065 (11)	−0.0209 (13)
N2	0.0347 (13)	0.0361 (14)	0.0325 (13)	−0.0015 (11)	−0.0033 (10)	−0.0171 (11)
C8	0.0427 (18)	0.046 (2)	0.0423 (18)	−0.0025 (15)	−0.0101 (14)	−0.0226 (16)
C19	0.0357 (15)	0.0329 (16)	0.0375 (16)	−0.0008 (12)	−0.0026 (12)	−0.0201 (13)
C5	0.0475 (18)	0.0317 (16)	0.0321 (15)	−0.0049 (14)	−0.0008 (13)	−0.0124 (13)
C18	0.0331 (15)	0.0352 (16)	0.0409 (17)	−0.0011 (13)	−0.0040 (12)	−0.0202 (14)
N4	0.0570 (19)	0.0462 (18)	0.0403 (16)	−0.0087 (15)	−0.0024 (13)	−0.0240 (14)
N1	0.0447 (17)	0.057 (2)	0.0392 (16)	−0.0019 (14)	0.0025 (13)	−0.0222 (15)
C13	0.054 (2)	0.054 (2)	0.056 (2)	−0.0044 (18)	−0.0046 (17)	−0.037 (2)
C15	0.0332 (16)	0.048 (2)	0.048 (2)	−0.0018 (15)	0.0086 (14)	−0.0184 (17)
C21	0.048 (2)	0.044 (2)	0.0414 (18)	−0.0037 (16)	0.0009 (15)	−0.0192 (16)
C3	0.073 (3)	0.061 (3)	0.0342 (18)	−0.024 (2)	0.0111 (18)	−0.0161 (18)
C23	0.078 (3)	0.055 (3)	0.0374 (19)	0.002 (2)	−0.0141 (19)	−0.0172 (18)
C12	0.077 (3)	0.063 (3)	0.054 (2)	−0.021 (2)	−0.003 (2)	−0.035 (2)
C1	0.048 (2)	0.087 (4)	0.051 (2)	0.005 (2)	0.0068 (18)	−0.030 (2)
C22	0.071 (3)	0.051 (2)	0.0328 (18)	−0.007 (2)	0.0037 (17)	−0.0168 (16)
C9	0.077 (3)	0.065 (3)	0.089 (4)	0.025 (3)	−0.046 (3)	−0.049 (3)
C24	0.052 (2)	0.063 (3)	0.045 (2)	0.0123 (19)	−0.0168 (17)	−0.0246 (19)
C2	0.066 (3)	0.082 (3)	0.046 (2)	−0.012 (3)	0.020 (2)	−0.028 (2)
C11	0.105 (4)	0.097 (4)	0.082 (4)	−0.007 (4)	−0.044 (3)	−0.054 (3)
C10	0.110 (5)	0.094 (5)	0.113 (5)	0.028 (4)	−0.079 (4)	−0.053 (4)
C14	0.053 (2)	0.047 (2)	0.048 (2)	−0.0031 (17)	0.0114 (16)	−0.0272 (17)
C4	0.058 (2)	0.056 (2)	0.0307 (17)	−0.0159 (19)	−0.0017 (15)	−0.0132 (16)

### Geometric parameters (Å, °)

**Table:**

Zn1—N2	2.151 (3)	N1—C1	1.335 (5)
Zn1—N6	2.177 (3)	C13—C14	1.388 (6)
Zn1—N1	2.202 (3)	C13—H13	0.9300
Zn1—Br1	2.3692 (7)	C15—C14	1.376 (6)
Zn1—Br2	2.3880 (6)	C15—H15	0.9300
N5—C13	1.330 (5)	C21—C22	1.388 (6)
N5—C17	1.339 (4)	C21—H21	0.9300
N3—C7	1.330 (4)	C3—C2	1.363 (7)
N3—C18	1.339 (4)	C3—C4	1.381 (6)
C7—C6	1.403 (5)	C3—H3	0.9300
C7—C8	1.487 (5)	C23—C24	1.372 (6)
C20—N6	1.336 (4)	C23—C22	1.372 (7)
C20—C21	1.392 (5)	C23—H23	0.9300
C20—C19	1.488 (4)	C12—C11	1.357 (8)
C16—C15	1.371 (5)	C12—H12	0.9300
C16—C17	1.392 (5)	C1—C2	1.392 (6)
C16—H16	0.9300	C1—H1	0.9300
C6—N2	1.344 (4)	C22—H22	0.9300
C6—C5	1.481 (5)	C9—C10	1.394 (7)
C17—C18	1.477 (4)	C9—H9	0.9300
N6—C24	1.351 (5)	C24—H24	0.9300
N2—C19	1.341 (4)	C2—H2	0.9300
C8—N4	1.333 (5)	C11—C10	1.359 (9)
C8—C9	1.379 (6)	C11—H11	0.9300
C19—C18	1.404 (4)	C10—H10	0.9300
C5—N1	1.334 (5)	C14—H14	0.9300
C5—C4	1.382 (5)	C4—H4	0.9300
N4—C12	1.338 (5)		
			
N2—Zn1—N6	73.75 (10)	C5—N1—Zn1	118.2 (2)
N2—Zn1—N1	72.96 (11)	C1—N1—Zn1	122.6 (3)
N6—Zn1—N1	146.71 (11)	N5—C13—C14	123.5 (4)
N2—Zn1—Br1	125.38 (8)	N5—C13—H13	118.3
N6—Zn1—Br1	102.12 (9)	C14—C13—H13	118.3
N1—Zn1—Br1	97.43 (10)	C16—C15—C14	119.5 (3)
N2—Zn1—Br2	118.61 (8)	C16—C15—H15	120.2
N6—Zn1—Br2	97.55 (9)	C14—C15—H15	120.2
N1—Zn1—Br2	97.76 (10)	C22—C21—C20	118.0 (4)
Br1—Zn1—Br2	115.93 (2)	C22—C21—H21	121.0
C13—N5—C17	117.3 (3)	C20—C21—H21	121.0
C7—N3—C18	120.4 (3)	C2—C3—C4	119.8 (4)
N3—C7—C6	119.5 (3)	C2—C3—H3	120.1
N3—C7—C8	116.7 (3)	C4—C3—H3	120.1
C6—C7—C8	123.8 (3)	C24—C23—C22	118.8 (4)
N6—C20—C21	122.2 (3)	C24—C23—H23	120.6
N6—C20—C19	114.2 (3)	C22—C23—H23	120.6
C21—C20—C19	123.5 (3)	N4—C12—C11	123.1 (4)
C15—C16—C17	118.4 (3)	N4—C12—H12	118.4
C15—C16—H16	120.8	C11—C12—H12	118.4
C17—C16—H16	120.8	N1—C1—C2	122.4 (4)
N2—C6—C7	117.2 (3)	N1—C1—H1	118.8
N2—C6—C5	114.1 (3)	C2—C1—H1	118.8
C7—C6—C5	128.7 (3)	C23—C22—C21	119.9 (4)
N5—C17—C16	123.0 (3)	C23—C22—H22	120.0
N5—C17—C18	115.8 (3)	C21—C22—H22	120.0
C16—C17—C18	120.9 (3)	C8—C9—C10	116.9 (5)
C20—N6—C24	118.6 (3)	C8—C9—H9	121.6
C20—N6—Zn1	117.8 (2)	C10—C9—H9	121.6
C24—N6—Zn1	122.1 (3)	N6—C24—C23	122.4 (4)
C19—N2—C6	121.7 (3)	N6—C24—H24	118.8
C19—N2—Zn1	118.5 (2)	C23—C24—H24	118.8
C6—N2—Zn1	119.7 (2)	C3—C2—C1	118.0 (4)
N4—C8—C9	123.8 (4)	C3—C2—H2	121.0
N4—C8—C7	114.3 (3)	C1—C2—H2	121.0
C9—C8—C7	121.9 (4)	C12—C11—C10	119.3 (4)
N2—C19—C18	117.6 (3)	C12—C11—H11	120.3
N2—C19—C20	113.7 (3)	C10—C11—H11	120.3
C18—C19—C20	128.6 (3)	C11—C10—C9	119.6 (5)
N1—C5—C4	121.3 (3)	C11—C10—H10	120.2
N1—C5—C6	114.6 (3)	C9—C10—H10	120.2
C4—C5—C6	124.1 (3)	C15—C14—C13	118.2 (3)
N3—C18—C19	118.8 (3)	C15—C14—H14	120.9
N3—C18—C17	115.5 (3)	C13—C14—H14	120.9
C19—C18—C17	125.6 (3)	C3—C4—C5	119.1 (4)
C8—N4—C12	117.2 (4)	C3—C4—H4	120.4
C5—N1—C1	119.2 (3)	C5—C4—H4	120.4
			
C18—N3—C7—C6	11.8 (5)	C7—C6—C5—C4	−6.9 (6)
C18—N3—C7—C8	−166.3 (3)	C7—N3—C18—C19	8.3 (5)
N3—C7—C6—N2	−20.0 (5)	C7—N3—C18—C17	−168.8 (3)
C8—C7—C6—N2	157.9 (3)	N2—C19—C18—N3	−20.2 (5)
N3—C7—C6—C5	159.1 (4)	C20—C19—C18—N3	158.3 (3)
C8—C7—C6—C5	−22.9 (6)	N2—C19—C18—C17	156.6 (3)
C13—N5—C17—C16	−1.5 (6)	C20—C19—C18—C17	−24.9 (6)
C13—N5—C17—C18	−176.1 (4)	N5—C17—C18—N3	149.6 (3)
C15—C16—C17—N5	−0.5 (6)	C16—C17—C18—N3	−25.1 (5)
C15—C16—C17—C18	173.9 (3)	N5—C17—C18—C19	−27.2 (5)
C21—C20—N6—C24	2.2 (6)	C16—C17—C18—C19	158.1 (4)
C19—C20—N6—C24	177.5 (3)	C9—C8—N4—C12	−1.2 (7)
C21—C20—N6—Zn1	−164.1 (3)	C7—C8—N4—C12	179.7 (4)
C19—C20—N6—Zn1	11.1 (4)	C4—C5—N1—C1	1.6 (6)
N2—Zn1—N6—C20	−3.2 (3)	C6—C5—N1—C1	179.8 (4)
N1—Zn1—N6—C20	−2.2 (4)	C4—C5—N1—Zn1	−176.4 (3)
Br1—Zn1—N6—C20	−126.8 (3)	C6—C5—N1—Zn1	1.9 (4)
Br2—Zn1—N6—C20	114.5 (3)	N2—Zn1—N1—C5	1.4 (3)
N2—Zn1—N6—C24	−169.0 (4)	N6—Zn1—N1—C5	0.4 (4)
N1—Zn1—N6—C24	−168.1 (3)	Br1—Zn1—N1—C5	126.2 (3)
Br1—Zn1—N6—C24	67.3 (3)	Br2—Zn1—N1—C5	−116.2 (3)
Br2—Zn1—N6—C24	−51.3 (3)	N2—Zn1—N1—C1	−176.5 (4)
C7—C6—N2—C19	7.8 (5)	N6—Zn1—N1—C1	−177.5 (4)
C5—C6—N2—C19	−171.5 (3)	Br1—Zn1—N1—C1	−51.7 (4)
C7—C6—N2—Zn1	−173.3 (2)	Br2—Zn1—N1—C1	65.9 (4)
C5—C6—N2—Zn1	7.5 (4)	C17—N5—C13—C14	2.1 (7)
N6—Zn1—N2—C19	−6.5 (3)	C17—C16—C15—C14	1.9 (6)
N1—Zn1—N2—C19	174.1 (3)	N6—C20—C21—C22	−2.2 (6)
Br1—Zn1—N2—C19	87.0 (3)	C19—C20—C21—C22	−177.1 (4)
Br2—Zn1—N2—C19	−96.3 (2)	C8—N4—C12—C11	−1.1 (7)
N6—Zn1—N2—C6	174.5 (3)	C5—N1—C1—C2	1.6 (8)
N1—Zn1—N2—C6	−4.9 (3)	Zn1—N1—C1—C2	179.5 (4)
Br1—Zn1—N2—C6	−92.0 (3)	C24—C23—C22—C21	0.5 (7)
Br2—Zn1—N2—C6	84.7 (3)	C20—C21—C22—C23	0.8 (6)
N3—C7—C8—N4	135.7 (4)	N4—C8—C9—C10	2.2 (9)
C6—C7—C8—N4	−42.3 (5)	C7—C8—C9—C10	−178.8 (6)
N3—C7—C8—C9	−43.4 (6)	C20—N6—C24—C23	−0.8 (7)
C6—C7—C8—C9	138.6 (5)	Zn1—N6—C24—C23	164.9 (4)
C6—N2—C19—C18	11.8 (5)	C22—C23—C24—N6	−0.5 (7)
Zn1—N2—C19—C18	−167.2 (2)	C4—C3—C2—C1	1.6 (8)
C6—N2—C19—C20	−167.0 (3)	N1—C1—C2—C3	−3.2 (8)
Zn1—N2—C19—C20	14.1 (4)	N4—C12—C11—C10	2.2 (10)
N6—C20—C19—N2	−16.2 (4)	C12—C11—C10—C9	−1.1 (12)
C21—C20—C19—N2	159.0 (3)	C8—C9—C10—C11	−1.0 (12)
N6—C20—C19—C18	165.2 (4)	C16—C15—C14—C13	−1.4 (6)
C21—C20—C19—C18	−19.6 (6)	N5—C13—C14—C15	−0.7 (7)
N2—C6—C5—N1	−5.9 (5)	C2—C3—C4—C5	1.3 (7)
C7—C6—C5—N1	174.9 (4)	N1—C5—C4—C3	−3.1 (6)
N2—C6—C5—C4	172.3 (4)	C6—C5—C4—C3	178.9 (4)

### Hydrogen-bond geometry (Å, °)

**Table:**

*D*—H···*A*	*D*—H	H···*A*	*D*···*A*	*D*—H···*A*
C11—H11···Br2^i^	0.93	2.88	3.791 (7)	166

Symmetry codes: (i) −*x*+2, −*y*+1, −*z*+2.
